# Long-term Cardiovascular Outcomes in Patients with Omicron COVID-19 and Elevated Cardiac Biomarkers: A Prospective Multicenter Cohort Study in Shanghai, China

**DOI:** 10.7150/ijms.112282

**Published:** 2025-06-09

**Authors:** Shun Yao, Yamei Xu, Zhonglei Xie, Shuai Yuan, Junqing Gao, Qianwei Chen, Kailei Shi, Zongjun Liu, Xiaotong Cui, Yanyan Wang, Yu Song, Xueting Han, Junbo Ge, Zhenju Song, Jingmin Zhou

**Affiliations:** 1 Department of Cardiology, Zhongshan Hospital, Fudan University, Shanghai Institute of Cardiovascular Diseases, China.; 2 Institutes of Biomedical Sciences Fudan University, Shanghai, China.; 3 Department of Cardiology, Putuo Hospital, Shanghai University of Traditional Chinese Medicine, Shanghai, China.; 4 Department of Cardiology, Huadong Hospital, Fudan University, Shanghai, China.; 5 Department of Emergency, Zhongshan Hospital, Fudan University, Shanghai, China.

**Keywords:** severe acute respiratory syndrome coronavirus 2 (SARS-CoV-2), coronavirus disease 2019 (COVID-19), Omicron variant, N-terminal pro-B-type natriuretic peptide (NT-proBNP), cardiac troponins T (cTnT)

## Abstract

**Background:** The long-term cardiovascular outcomes of SARS-CoV-2 omicron-infected patients remain unclear. This study aimed to evaluate acute and long-term cardiovascular risks in hospitalized omicron-infected patients with elevated cardiac biomarkers.

**Methods:** We included 3012 patients hospitalized in Shanghai, China, between December 1, 2022, and January 31, 2023. Participants were stratified into four groups based on cardiac troponin T (cTnT) and N-terminal pro-B-type natriuretic peptide (NT-proBNP) levels. Major adverse cardiovascular events (MACEs), all-cause death, cardiovascular death, and cardiovascular-related rehospitalization were evaluated over a 12-month follow-up.

**Results:** Patients with elevated cTnT and high NT-proBNP had significantly higher risks of MACEs (HRadj=2.85, 95% CI 1.58-5.12), all-cause death (HRadj=5.56, 95% CI 1.51-20.52), cardiovascular death (HRadj=11.97, 95% CI 1.40-102.46), and cardiovascular-related rehospitalization (HRadj=2.38, 95% CI 1.28-4.42). The finding of Subgroup analyses indicated the risk of MACEs were independent of age, gender, hypertension, coronary artery disease, acute coronary syndrome, or heart failure.

**Conclusions:** Elevated cTnT and NT-proBNP levels during the acute phase of omicron infection predict a substantially increased risk of adverse cardiovascular outcomes within 12 months.

## Introduction

The coronavirus disease 2019 (COVID-19) pandemic is still ongoing across the globe, and more than 777 million people worldwide have been infected with severe acute respiratory syndrome coronavirus 2 (SARS-CoV-2) up to February 2, 2025, according to the WHO COVID-19 record 1. Increasing evidence suggests that people who have recovered from COVID-19 may experience continuous symptoms in multiple organs and systems 2, 3. Some patients represent increased risks of cardiovascular diseases, including cerebrovascular diseases, arrhythmias, ischemic and non-ischemic heart diseases, pericarditis, myocarditis, heart failure (HF), and thromboembolic disorders 4, 5. Since 2022, the omicron variant has gradually become the predominant strain of the SARS-CoV-2 in China 6, 7. Related research indicates that the clinical significance of infections caused by the omicron variant differs from that of the other SARS-CoV-2 variants. The omicron variant exhibits a higher transmissibility and an enhanced ability to evade immunity than the delta variant 8-10. However, limited studies have been reported on the long-term adverse cardiovascular outcomes of omicron variant-infections.

N-terminal pro-B-type natriuretic peptide (NT-proBNP) and cardiac troponin-T (cTnT) are biomarkers commonly used to diagnose HF and myocardial injury and to predict adverse cardiovascular events. Previous studies have shown that elevation in cardiac troponin or NT-proBNP was associated with a higher risk of mortality in hospitalized patients with COVID-19 11-14. Elevated NT-proBNP and cTnT levels could better predict all-cause mortality in COVID-19 patients post-discharge 15. However, these studies were not conducted on the prevalence of the omicron variant and only focused on all-cause deaths, not including other cardiovascular events such as cardiovascular death, acute myocardial infarction, stroke, HF, and cerebrovascular diseases. Hence, further study is required to determine the long-term effects of the omicron variant on multiple cardiovascular events.

Therefore, this study aimed to investigate whether the combination of NT-proBNP and cTnT levels is associated with cardiovascular outcomes in hospitalized patients infected with the SARS-CoV-2 omicron variant.

## Methods

### Study design

The present study was a multicenter observational prospective cohort study approved by the Ethics Committee of Zhongshan Hospital (B2023-062), Fudan University, Shanghai, China. The inclusion criteria were patients aged ≥15 years with SARS-CoV-2 infection confirmed by reverse transcriptase-polymerase chain reaction or positive IgM serology before or during the index hospitalization, regardless of history of cardiovascular disease (CVD) between December 1, 2022, and January 31, 2023. This multicenter study involved three hospitals in the Shanghai Metropolitan Region: Zhongshan Hospital of Fudan University, Huadong Hospital Affiliated with Fudan University, and Putuo Hospital Affiliated with Shanghai University of Traditional Chinese Medicine. This study enrolled patients who signed informed consent forms and underwent NT-proBNP and cTnT tests on admission and during hospitalization when clinically indicated. For analysis, we used the peak (highest) value during hospitalization.

### Data collection

Trained clinical staff and data managers recorded demographic data, comorbidities, laboratory findings, treatments, and outcomes using standardized forms. This included medical history, vital signs, clinical laboratory data, medication, non-drug treatment, and in-hospital adverse events, such as hypoxemia, respiratory failure, severe pneumonia, sepsis, cardiac arrest, and shock. Transthoracic echocardiography was performed based on clinical indications and at the discretion of the attending physician. Serum cTnT and NT-proBNP levels were measured using the Roche Cobas 801 analyzer with an electrochemiluminescence immunoassay (ECLIA) method.

### Outcome assessments

After discharge, patients were advised to schedule follow-up appointments with the outpatient department unless they could not attend in person; in this case, our staff contacted them by phone. The primary outcome was MACEs, which was defined as cardiovascular-related death (CV death), acute myocardial infarction (AMI), stroke, or acute HF (new-onset or worsening). Secondary outcomes included all-cause death, CV death, and cardiovascular-related rehospitalization (CV-related rehospitalization). The primary outcome was MACEs, which was defined as cardiovascular-related death, acute myocardial infarction (AMI), stroke, or acute heart failure (new-onset or worsening). While cardiovascular death was included as a component of MACE, we also reported it separately to enhance the understanding of its specific contribution to the composite outcome, consistent with prior studies 16. The last date for follow-up assessments was on January 31, 2024.

### Statistical analysis

To enhance specificity, we defined patients with NT-proBNP levels above the upper quartile (848.3 pg/mL) as the high NT-proBNP group, and those with levels at or below this threshold as the low NT-proBNP group, as used in prior biomarker-based stratification studies 17. Similarly, we defined patients with normal cTnT levels (<0.014 pg/mL) and elevated cTnT levels (≥ 0.014 pg/mL) according to the 99th percentile upper-reference limit (URL). For continuous variables, normality was assessed via the Shapiro‒Wilk test. Means with standard deviations were reported for normally distributed data, whereas medians with interquartile ranges were reported for data that deviated from normality. Depending on the data distribution, between-group comparisons were performed using ANOVA or the Kruskal-Wallis test. Categorical variables were expressed as counts and percentages, and subsequent comparisons were performed using Chi-squared tests or Fisher's exact tests as appropriate. Analyses were performed by including patients with available data.

Crude event rates were reported for all outcomes. The cumulative incidence of events was assessed using the Kaplan-Meier method, and the log-rank test was used to examine differences in the time-to-event distributions. The median follow-up duration was estimated using the reverse Kaplan-Meier method. Hazard ratios (HRs) with 95% confidence intervals (CIs) were calculated in the multivariate-adjusted Cox proportional hazards model. Variables included in multivariate-adjusted models were selected by the combination of clinical relevance, a univariate relationship with the outcome, and potential association with outcome published previously: age, sex, body mass index, left ventricular ejection fraction, previous diagnoses of coronary artery disease (CAD), HF, hypertension, diabetes, stroke, chronic kidney disease, and malignant tumor 18. The number of available events was also considered to ensure parsimony and avoid the problem of overfitting the final model. Proportional hazard assumptions were assessed using the Schoenfeld residuals test, and no significant violations of this assumption were found (P >0.05 for all models). A refined sub-distribution hazard model, the Fine & Gray model, was applied to estimate the secondary endpoint, accounting for potential competing risks from all-cause deaths 19.

Subgroup analyses were also performed to examine the possible influence of the vital factors on the primary outcome according to age, sex, and previous diagnoses of CAD and hypertension. Age 65 was selected as the cutoff for subgroup analysis to distinguish elderly from non-elderly patients, consistent with common clinical and epidemiological definitions in cardiovascular research 20, 21. Interactions between the treatment effect and these prespecified grouping factors (interaction P values) were calculated by incorporating an interaction term (subgroup variable × treatment variable) within the Cox model.

Statistical analyses were conducted using R software version 3.6.0 (R Foundation for Statistical Computing, Vienna, Austria) and SPSS 27.0. A two-tailed alpha level of <0.05 was considered to indicate statistical significance. No multiple comparison adjustments were conducted; thus, the outcomes of secondary endpoints, subgroups, and sensitivity analyses should be regarded as exploratory with susceptibility to type 1 error.

## Results

### Baseline demographic characteristics

**Figure 1:**
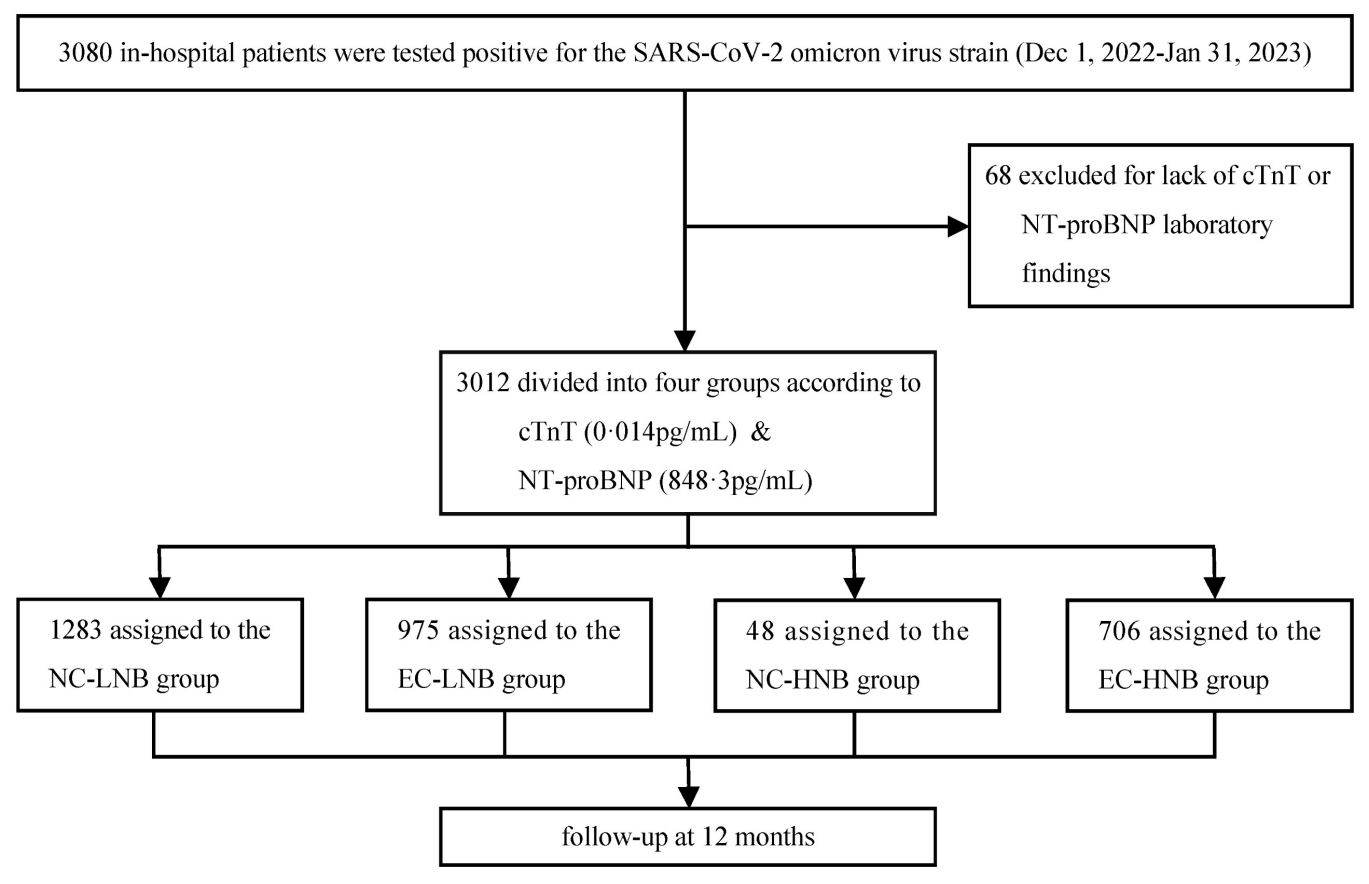
The diagram illustrates the flow of patient inclusion in this cohort. The number of patients analyzed for MACE outcomes reflects those with completed follow-up; some patients were lost during the 12-month observation period. The NC-LNB group, the normal cTnT and low NT-proBNP group; The EC-LNB group, the elevated cTnT and low NT-proBNP group; The NC-HNB group, the normal cTnT and high NT-proBNP group; The EC-HNB group, the elevated cTnT and high NT-proBNP group. cTnT, cardiac troponin-T; NT-proBNP, N-terminal pro-B-type natriuretic peptide.

A total of 3,080 patients who were hospitalized with a diagnosis of COVID-19 between December 1, 2022, and January 31, 2023, were included in this study. After the exclusion of 68 patients with missing cTnT or NT-proBNP data, a total of 3012 patients were included in the subsequent analysis, and 2785 patients completed the follow-up (Figure 1). All patients were divided into four groups according to the threshold level of cTnT (0.014 pg/mL) and upper quartile value (848.3 pg/mL) of NT-proBNP: (i) the NC-LNB group: the normal cTnT and low NT-proBNP group (n=1283; 42.6%); (ii) the EC-LNB group: the elevated cTnT and low NT-proBNP group (n=975; 32.4%); (iii) the NC-HNB group: the normal cTnT and high NT-proBNP group (n=48; 1.6%); (iv) the EC-HNB group: the elevated cTnT and high NT-proBNP group (n=706; 23.4%) (Figure 1). The overall cohort had a median follow-up duration of 7.4 months, primarily due to the inclusion of patients who died during hospitalization and those lost to follow-up after discharge. The baseline demographic characteristics of the patients are presented in Table 1. In the overall cohort, the median age was 66 (inter-quartile range, 57-73) years, and 35.0% (1055/3012) were female. Patients in the EC-HNB group exhibited older age, lower body mass index and diastolic blood pressure, and higher pulse rates. In addition, the EC-HNB group also showed a more pronounced burden of comorbidities, manifested by a higher prevalence of AECOPD, pneumonia, diabetes, chronic kidney disease, ischaemic stroke, myocarditis, and malignancy. The peak levels of cTnT and NT-proBNP in the EC-HNB group were also significantly higher than those in the single biomarker-elevated group (Table 1). Laboratory tests revealed that the EC-HNB group had the heaviest inflammatory burden, characterized by the highest white blood cell count, CRP, IL-6, and PCT levels. In addition, mild elevations in AST and creatinine suggested a moderate impairment of liver and kidney function in this group. Echocardiography revealed impaired systolic and diastolic function of the left ventricle of these patients.

### Primary endpoint outcomes

**Figure 2:**
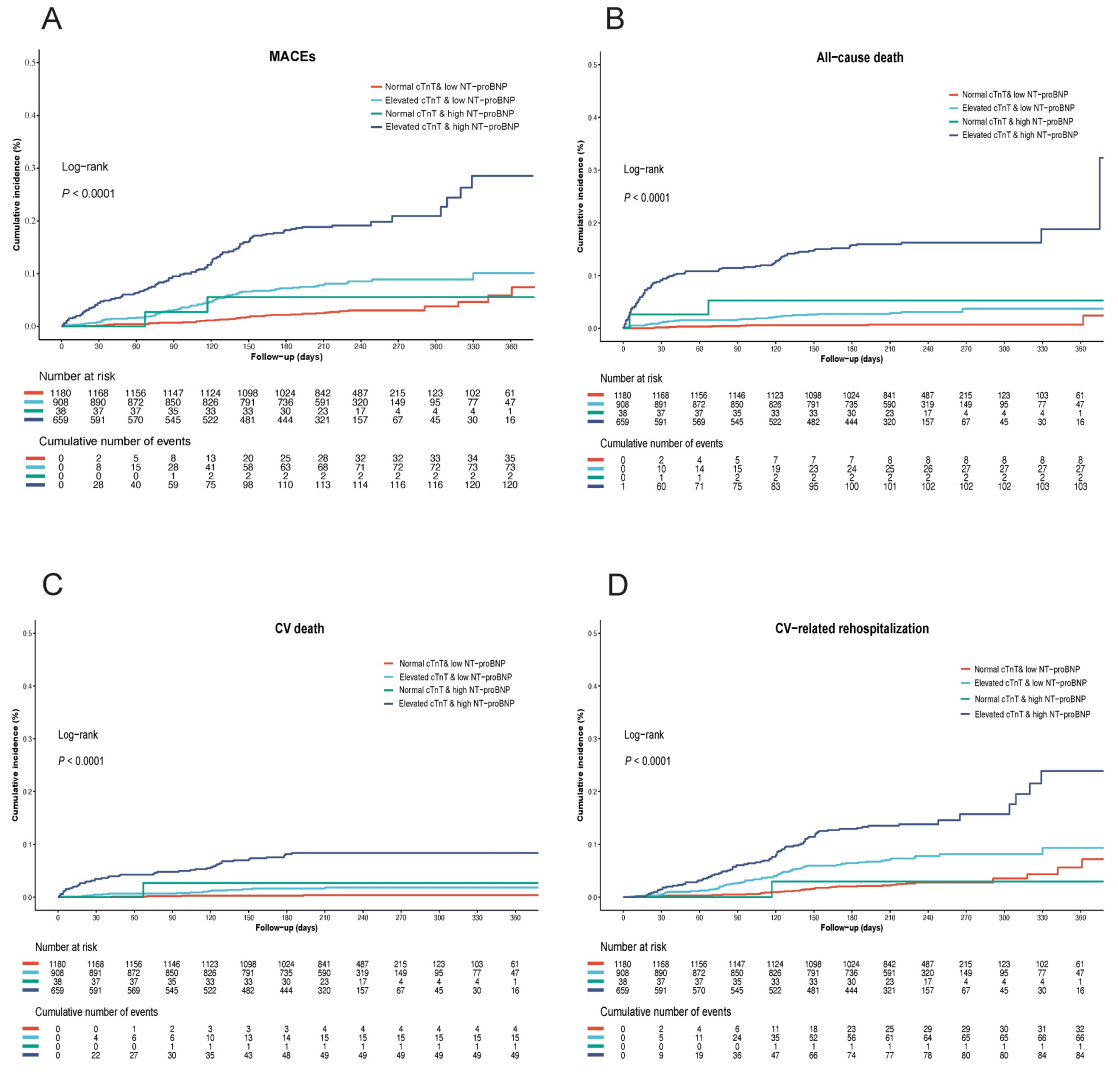
Kaplan-Meier curves stratified by the level of cTnT and NT-proBNP for the cumulative incidence of (A) MACEs, (B) all-cause death, (C) cardiovascular death, (D) cardiovascular-related rehospitalization in the overall cohort. MACEs, major adverse cardiovascular events.

Among the overall cohort, a total of 227 patients (7.5%) dropped out during the follow-up, and 232 patients (8.3%) had MACEs within 1-year. Specifically, 103 patients from the NC-LNB group, 67 from the EC-LNB group, 10 from the NC-HNB group, and 47 from the EC-HNB group were lost to follow-up, resulting in 1180, 908, 38, and 659 patients respectively being included in the outcome analyses. After adjusting for age, sex, body mass index, left ventricular ejection fraction, and histories of CAD, HF, hypertension, diabetes, stroke, chronic kidney disease, and malignancy, the EC-HNB group remained significantly associated with the highest risk of MACEs. Specifically, 120 out of 659 patients (18.2%) in the EC-HNB group experienced MACEs, compared with 37 out of 1180 (3.1%) in the NC-LNB group, 73 out of 908 (8.0%) in the EC-LNB group, and 2 out of 38 (5.3%) in the NC-HNB group (P < 0.001 by log-rank test; Table 2 and Figure 2A). In the subgroup of patients discharged alive (n=2946) (Figure S1), 211 (7.8%) experienced MACEs within 1 year. The EC-HNB group remained associated with the highest MACE risk (16.7%), compared with 8.0% in the EC-LNB group, 5.4% in the NC-HNB group, and 3.1% in the NC-LNB group (P < 0.001; Table S1, Figure S2). This association remained significant after multivariable adjustment (adjusted HR for EC-HNB vs. NC-LNB: 2.58 [95% CI, 1.42-4.70]; P < 0.001; Table S1). To assess whether the presence of acute coronary syndrome (ACS) or HF affected the prognostic significance of elevated cTnT and high NT-proBNP, the total cohort was further divided into two subgroups: those with or without the presence of ACS or HF (ACS/HF). The results showed that the EC-HNB group tended to have the highest rate of MACEs, followed by the EC-LNB group, the NC-HNB group, and the NC-LNB group regardless of ACS/HF status (Table S2, Figure S2A, and Figure S3A). Even after adjustment for potential confounders, the association between elevated cTnT and high NT-proBNP and increased MACEs remained statistically significant when the NC-LNB group was used as a reference (adjusted HR for elevated cTnT and high NT-proBNP, 2.85 [95% CI, 1.58-5.12], P < 0.001; Table 2).

**Figure 3:**
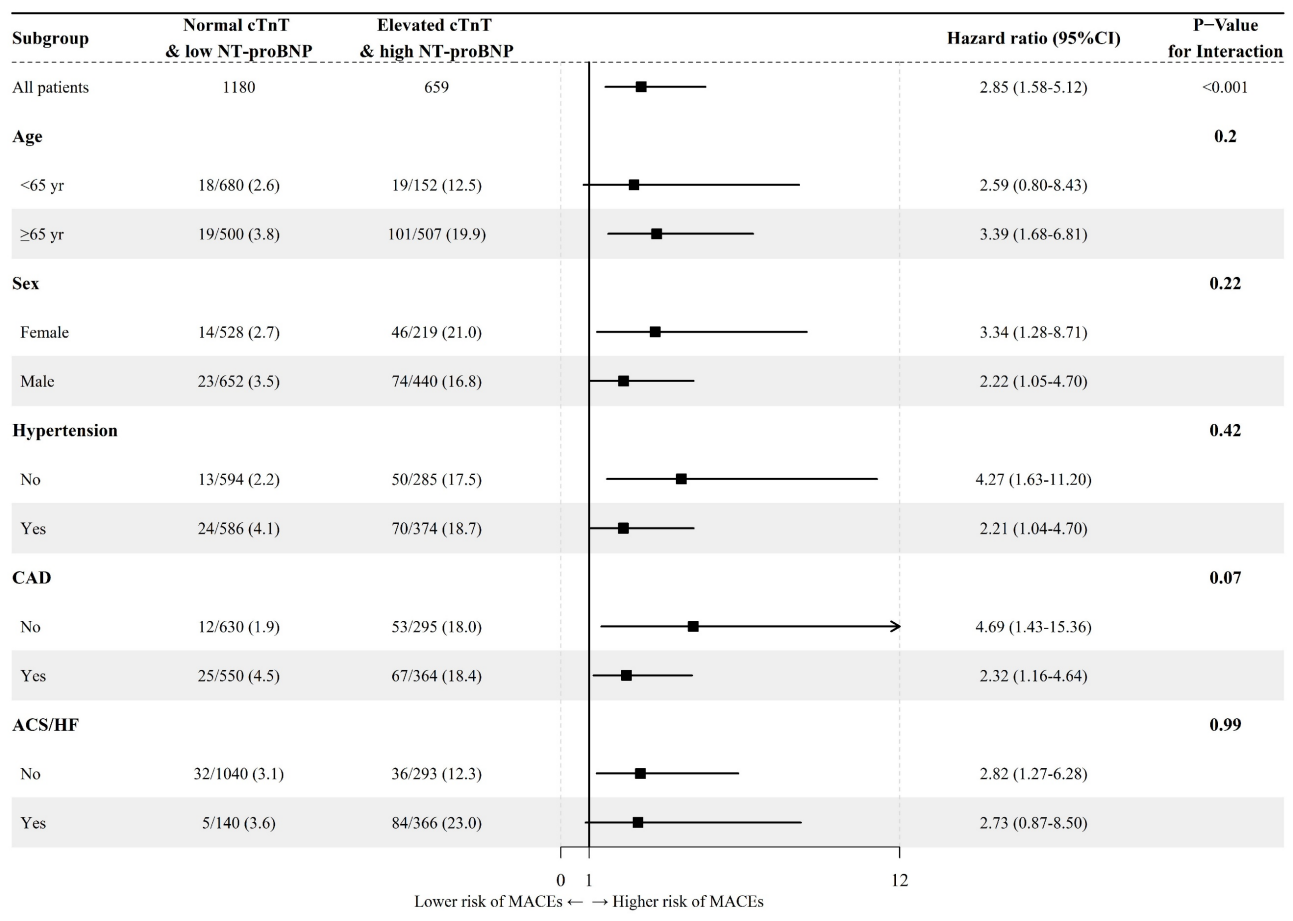
Forest plot of prespecified subgroup analyses of the MACEs in the overall cohort. ACS, acute coronary syndrome, CAD, coronary artery disease; CI, confidence intervals; HF, heart failure; HR, hazard ratio.

Due to the relatively small number of patients in the single marker group, especially in the NC-HNB group, subgroup analyses were only conducted comparing the EC-HNB group and the NC-LNB group. The result indicated no significant interaction between the elevation of cardiac markers and other vital factors. That is, the relationship between the combination of elevated cTnT and high NT-proBNP and adverse outcomes was not influenced by age, sex, and whether there was a history of hypertension or CAD and the presence of ACS or HF (Figure 3). To further evaluate whether systemic inflammation influenced cardiovascular outcomes, we conducted a subgroup analysis based on inflammatory marker levels. In a subgroup analysis stratified by the upper limits of normal level of CRP levels (available in 2748 patients), the EC-HNB group consistently had the highest MACEs incidence regardless of CRP status (< or ≥ 3.0 mg/L). No significant interaction was found between CRP level and cardiac biomarker-defined groups (Figure S5; Table S2).

### Secondary endpoint outcomes

Analysis of secondary endpoint outcomes revealed a significant increase in all-cause death, CV death, and CV-related rehospitalization in the EC-HNB group (Figure 2B-D). A similar trend was also observed in both the ACS/HF and the non-ACS/HF groups (Figure S2B-D and Figure S3B-D). The association between the EC-HNB group and the higher risk of the secondary endpoint outcomes was further confirmed by the multivariate-adjusted analysis using the NC-LNB group as a reference (adjusted HR for all-cause death, 5.56 [95% CI, 1.51, 20.52], P = 0.01; adjusted HR for CV death, 11.97 [95% CI, 1.40, 102.46], P = 0.023; adjusted HR for CV-related rehospitalization, 2.38 [95% CI, 1.28, 4.42], P = 0.006; Table 2).

### In-hospital treatments and adverse events

Overall, the use of drugs for COVID-19, including Paxlovid and Azvudine, was relatively low in this cohort (Table 3). In line with the higher comorbidity and inflammatory burden, patients in the EC-HNB group were more likely to be prescribed glucocorticoids, inotropes, vasoactive drugs, oral anticoagulants, enoxaparin sodium, and loop diuretics. Non-pharmacological treatments showed similar trends presented as higher rates of nasal cannula oxygen therapy, mask oxygen therapy, invasive mechanical ventilation, ECMO, and IABP. Correspondingly, the length of hospital stay for patients in this group was significantly longer than that for patients in the other groups.

Despite the aggressive therapeutic measures, the incidence of in-hospital adverse events in this group of patients remained significantly higher than that in patients with a single marker elevation or those with no marker elevation (Table 3). Adverse events, including hypoxemia, respiratory failure, severe pneumonia, sepsis, cardiac arrest, and shock, were predominantly concentrated in patients with elevated cTnT and high NT-proBNP. Among the 66 patients who experienced in-hospital mortality, 59 were in the EC-HNB group.

## Discussion

This study demonstrated that elevated levels of cTnT and high NT-proBNP were significantly associated with increased risks of MACEs, mortality, and rehospitalization in patients infected with COVID-19. These findings suggest that assessing these two cardiac biomarkers may be valuable for predicting long-term adverse cardiovascular outcomes in these patients.

Notably, COVID-19 might coexist with humans for an extended period in the coming years and increase the risk of acute and post-acute adverse cardiovascular outcomes 22, 23. The continuous evolution of the SARS-CoV-2 virus into new variants with greater infectivity poses unpredictable health risks 24, 25. Hence, it is imperative to consistently monitor the diverse impacts of emerging variant strains on cardiovascular events. Presently, there is insufficient short- and long-term research on the incidence of multiple cardiovascular events in East Asian populations with COVID-19. Thus, our study was conducted to fill the gap in this research field and provide valuable insights into this aspect.

Our study is the first prospective cohort study using combined cTnT and NT-proBNP biomarkers for long-term cardiovascular risk assessments in COVID-19 patients. When COVID-19 patients have adverse cardiovascular conditions, the predictive value of commonly used cardiac biomarkers for adverse cardiovascular outcomes may be limited. Patients with COVID-19 often exhibit a state of systemic inflammation, leading to a disturbance in the internal environment 13, which implies that the levels of biomarkers for myocardial injury are influenced by various factors such as infection, hypoxia, and renal function 26. Inflammatory factors within the body of COVID-19 patients, such as interleukin-6, may increase NT-proBNP 27. Moreover, a consistent elevation in inflammatory factor levels in COVID-19 patients aligns with increased NT-proBNP levels. These findings suggest that using the established NT-proBNP threshold to diagnose HF may not accurately evaluate cardiac pressure and volume load in COVID-19 patients, let alone predict the risk of cardiovascular events. Therefore, it is necessary to consider the potential possibility of 'false positive' myocardial injury in COVID-19 patients. For this reason, aiming to enhance the specificity of predicting the value of the risk of cardiovascular events, we categorized COVID-19 patients into high- and low-NT-proBNP groups using the upper quartile of NT-proBNP as a threshold and divided patients into normal and elevated cTnT groups using the 99th percentile upper reference limit as the threshold of cTnT. We further combined the groups of cTnT and NT-proBNP levels as a new approach for evaluating the predictive power for exploring the association between cardiac biomarkers and long-term cardiovascular risk in COVID-19 patients.

Our findings of the primary endpoint outcome demonstrated that COVID-19 patients with elevated cTnT levels had a higher prevalence of baseline cardiovascular conditions, such as atrial fibrillation, myocarditis, and heart failure. However, multivariable Cox regression analysis confirmed that elevated cTnT remained independently associated with an increased risk of MACEs after adjusting for these comorbidities and other confounding factors. Furthermore, patients with elevated levels of both cTnT and NT-proBNP exhibited the poorest cardiovascular prognosis, with an approximately three-fold higher risk of MACEs compared to those with normal cTnT and low NT-proBNP. A previous retrospective study reported that COVID-19 patients with elevated NT-proBNP (>1500 pg/mL) had a higher risk of in-hospital mortality (2.74 higher odds), and patients with low cTnT (<0.04 ng/mL) and high cTnT (≥0.04 ng/mL) exhibited an increased risk of in-hospital mortality (1.68 and 3.41 higher odds, respectively), compared to those with normal cTnT, additionally, patients with elevated levels of both cTnT and NT-proBNP had the worst outcomes, with a 6.10-fold higher risk of death 16. O'Donnell et al. reported that patients with higher levels of NT-proBNP have a higher risk of in-hospital mortality, specifically, for every two-fold increase in NT-proBNP levels, the risk of death increases by 21%. For patients with NT-proBNP levels above 2385 pg/mL, the risk of death is nearly seven times higher 19. Similarly, Majure et al. revealed that compared to COVID-19 patients with normal cardiac troponin I (cTnI), those with mild and severe cTnI elevation had a 1.06-fold and 3.51-fold increased risk of in-hospital death, respectively 28. These previous studies were limited by their retrospective nature, which only analyzed the association between NT-proBNP or cTnT and in-hospital mortality.

Elderly patients or patients with comorbidities such as ACS and HF typically have elevated levels of cTnT and NT-proBNP 29, 30, which may interfere with our findings. We conducted subgroup analyses to further validate whether factors such as age, sex, hypertension, CAD, ACS, or HF could have influenced the association between elevated cTnT and NT-proBNP levels and long-term cardiovascular outcomes. Notably, the prognostic value of cardiac biomarker elevation remained consistent across all subgroups. In addition, stratification by CRP levels, as a marker of systemic inflammation, showed similar results. The predictive association between biomarker-defined risk groups and MACEs was not significantly modified by baseline inflammatory status, suggesting robustness of our findings across varying clinical conditions. To further confirm this conclusion, we generated Kaplan-Meier curves for MACEs, all-cause death, CV death, and CV-related rehospitalization in patients with or without ACS/HF. The results indicated that patients with elevated cTnT and NT-proBNP had the highest risk of MACEs, regardless of whether they had ACS/HF. These findings from the subgroup analyses were consistent with those from the overall cohort study.

For secondary endpoint outcomes, COVID-19 patients with elevated cTnT and high NT-proBNP levels had the highest risk of all-cause death, CV death, and CV-related rehospitalizations. Compared to those in the normal cTnT and low NT-proBNP group, the elevated cTnT and high NT-proBNP group had approximately 6-fold higher odds of risk of all-cause death, and a 12-fold higher odds of risk of CV death, which is consistent with previous studies 13, 15, 31-34. These studies suggest that patients with higher levels of cTnT and NT-proBNP tended to have the worst cardiovascular outcomes. This finding implies that elevated cTnT and high NT-proBNP levels may be capable of predicting the risk of all-cause mortality, CV death, and CV-related rehospitalization. All-cause mortality was chosen as a robust endpoint for long-term risk assessment, considering the multifactorial nature of death in COVID-19 patients. This approach is supported by large cohort studies evaluating cardiac biomarkers such as troponin and natriuretic peptides 15, 33, 35.

The present study has several strengths: (i) our study is a multicenter, prospective cohort study with multiple cardiovascular endpoints, focusing on the cardiovascular prognosis of Chinese patients following COVID-19 Omicron infection, thereby filling a gap in research on the correlation between omicron variants and cardiovascular events in China. (ii) we combined two cardiac biomarkers and stratified patients based on the normal threshold level of cTnT and the upper quartile value of NT-proBNP, which enhanced the specificity of predicting adverse cardiovascular outcomes, and the identification of COVID-19 patients at higher risk of cardiovascular events, thus, this approach can facilitate early intervention, and contribute to justification for medical resource allocation; (iii) this study undertook a subgroup analysis to derive a robust conclusion, demonstrating that the increased risk of MACEs in patients with elevated cTnT and high NT-proBNP remains consistent, irrespective of age, sex, prior hypertension, prior CAD, or the presence of ACS or HF.

This study has several limitations: (i) the study was conducted during the outbreak of the COVID-19 omicron variant in China, where all hospitalized patients were COVID-19 positive, and therefore, a separate uninfected control group was not available for our research. To address this issue, we conducted a correlation analysis by grouping COVID-19 patients based on four levels of cTnT and NT-proBNP, ensuring that our research conclusions were not compromised by the absence of an uninfected control group; (ii) the sample size in our study, especially in the NC-HNB group, was limited, potentially increasing sampling errors, thus, it is imperative to conduct larger-scale prospective cohort studies involving diverse ethnic populations and including control groups to validate our research findings in the future; (iii) the missing data of 68 patients are due to their refusal to undergo plasma cTnT and NT-proBNP tests during hospitalization. Although a small proportion of patients (7.5%) were lost to follow-up, the baseline characteristics of these individuals were similar to those who completed the study, indicating a low likelihood of attrition bias influencing the primary outcomes; (iv) we were unable to obtain data on other lifestyle factors and daily medication records (including anticoagulant and antiplatelet medications) of patients during the post-discharge follow-up period, which could impact their cardiovascular outcomes; and (v) Our study results cannot indicate a causal association.

## Conclusions

In summary, the patients infected with the COVID-19 Omicron variant who have elevated levels of cTnT and NT-proBNP have a substantially higher risk of MACEs, mortality, and rehospitalization. Monitoring the levels of cTnT and NT-proBNP may provide an early assessment of cardiovascular prognosis in COVID-19 patients, guiding clinical decisions and medical resource allocation. For patients with elevated cTnT and NT-proBNP levels, it is necessary to provide rehabilitation support and arrange cardiovascular outpatient follow-up after the acute phase, which may reduce their risk of future adverse cardiovascular events. Our study provides a new research potential for investigating the association between similar viral outbreaks and adverse cardiovascular outcomes, which may be of great clinical significance in future studies of this nature.

## Supplementary Material

Supplementary figures and tables.

## Figures and Tables

**Table 1:** Baseline characteristics of patients according to the level of cTnT and NT-proBNP

Variables	No. of patients with available data	Overall	Normal cTnT & low NT-proBNP	Elevated cTnT & low NT-proBNP	Normal cTnT & high NT-proBNP	Elevated cTnT & high NT-proBNP	P-Value
		N=3012	N=1283	N=975	N=48	N=706	
Age, yr*		66 [57, 73]	62 [54, 69]	67 [59, 74]	66 [55, 72]	73 [66, 80]	<0.001
Female*		1055 (35.0)	579 (45.1)	213 (21.8)	26 (54.2)	237 (33.6)	<0.001
Body mass index, kg/m^2^	2876	24.4 [22.3, 26.6]	24.3 [22.4, 26.4]	24.8 [22.8, 27.3]	24.4 [22.0, 27.0]	23.7 [21.6, 26.0]	<0.001
Currently smoking	1690	699 (41.4)	253 (37.5)	306 (44.5)	4 (40.0)	136 (42.9)	0.060
Currently drinking	1659	177 (10.7)	70 (10.6)	66 (9.6)	3 (33.3)	38 (12.6)	0.083
Systolic pressure, mmHg	2986	130 [120, 140]	130 [120, 139]	131 [121, 143]	123 [108, 132]	130 [117, 144.5]	<0.001
Diastolic pressure, mmHg	2979	78 [70, 84]	79 [72, 85]	78 [70, 84]	77 [70.5, 80.8]	75 [68, 84]	<0.001
Fever*		204 (6.8)	106 (8.3)	45 (4.6)	3 (6.2)	50 (7.1)	0.006
Temperature, ℃	2996	36.5 [36.4, 36.7]	36.5 [36.3, 36.7]	36.5 [36.4, 36.7]	36.5 [36.5, 36.8]	36.5 [36.4, 36.8]	0.001
Respiratory rate, per min	2996	20 [18, 20]	20 [18, 20]	20 [18, 20]	20 [20, 20]	20 [18, 20]	<0.001
Pulse rate, bpm	2996	78 [70, 84]	78 [70, 83]	75 [70, 82]	79 [70, 90]	80 [70, 88]	<0.001
Length of hospital stay, day	3010	3 [2, 5]	3 [2, 4]	3 [2, 5]	4 [2.5, 6.5]	7 [3.5, 11.5]	<0.001
Complications							
AECOPD*		24 (0.8)	4 (0.3)	10 (1.0)	0 (0.0)	10 (1.4)	0.034
Pneumonia*		358 (11.9)	79 (6.2)	87 (8.9)	8 (16.7)	184 (26.1)	<0.001
ACS*		626 (20.8)	119 (9.3)	303 (31.1)	2 (4.2)	202 (28.6)	<0.001
History of CAD*		1764 (58.6)	601 (46.8)	760 (77.9)	9 (18.8)	394 (55.8)	<0.001
Heart failure*		350 (11.6)	44 (3.4)	61 (6.3)	17 (35.4)	228 (32.3)	<0.001
Hypertension*		1711 (56.8)	643 (50.1)	649 (66.6)	22 (45.8)	397 (56.2)	<0.001
Diabetes*		792 (26.3)	245 (19.1)	309 (31.7)	9 (18.8)	229 (32.4)	<0.001
Hyperlipidemia*		370 (12.3)	183 (14.3)	141 (14.5)	2 (4.2)	44 (6.2)	<0.001
Atrial fibrillation*		374 (12.4)	132 (10.3)	59 (6.1)	23 (47.9)	160 (22.7)	<0.001
Chronic kidney disease*		171 (5.7)	8 (0.6)	55 (5.6)	1 (2.1)	107 (15.2)	<0.001
Ischaemic stroke*		190 (6.3)	50 (3.9)	72 (7.4)	2 (4.2)	66 (9.3)	<0.001
Cardiomyopathy*		90(3.0)	12(0.9)	22(2.3)	4(8.3)	52(7.4)	<0.001
Myocarditis*		22 (0.7)	1 (0.1)	10 (1.0)	0 (0.0)	11 (1.6)	<0.001
Valvular heart disease*		130 (4.3)	30 (2.3)	32 (3.3)	8 (16.7)	60 (8.5)	<0.001
Pacemaker*		82 (2.7)	21 (1.6)	23 (2.4)	3 (6.2)	35 (5.0)	<0.001
Pulmonary hypertension*		54 (1.8)	13 (1.0)	9 (0.9)	6 (12.5)	26 (3.7)	<0.001
Pulmonary embolism*		24 (0.8)	6 (0.5)	6 (0.6)	0 (0.0)	12 (1.7)	0.039
Malignant tumor*		89 (3.0)	25 (1.9)	32 (3.3)	0 (0.0)	32 (4.5)	0.007
Laboratory fingdings							
Peak value of cTnT, pg/ml	3012	0.017 [0.007, 0.062]	0.007 [0.004, 0.009]	0.036 [0.020, 0.112]	0.010 [0.007, 0.011]	0.093 [0.034, 0.553]	<0.001
Peak value of NT-proBNP, pg/ml	3012	178.5 [59.6, 848.5]	66.2 [33.2, 136.0]	206.0 [87.6, 394.5]	1291.0 [1047.0, 1873.2]	2597.0 [1448.0, 5915.2]	<0.001
Red blood cell, ×10^12^/L	3006	4.360 [3.970, 4.728]	4.410 [4.110, 4.760]	4.400 [3.990, 4.760]	4.385 [4.090, 4.710]	4.135 [3.600, 4.590]	<0.001
White blood cell, ×10⁹/L	3006	6.280 [5.230, 7.680]	5.970 [5.060, 7.140]	6.370 [5.390, 7.685]	5.880 [4.907, 7.330]	6.935 [5.447, 8.755]	<0.001
Lymph, ×10⁹/L	3008	1.600 [1.200, 2.100]	1.800 [1.400, 2.200]	1.700 [1.300, 2.100]	1.700 [1.300, 2.100]	1.200 [0.800, 1.600]	<0.001
Platelet, ×10⁹/L	3006	201.0 [163.0, 242.0]	208.0 [174.0, 245.0]	200.0 [163.0, 241.0]	191.5 [167.0, 222.2]	188.0 [146.0, 237.0]	<0.001
Alanine transaminase, IU/L	3008	20 [14, 30]	19 [13, 28]	20 [15, 30]	24.5 [18.8, 40]	20 [14, 34]	<0.001
Aspartate transferase, IU/L	2742	21 [16, 27]	19 [16, 23]	21 [17, 27]	24 [20.5, 30]	25 [18, 42.8]	<0.001
Creatinine, μmol/L	2827	80 [67, 95]	73 [63, 85]	82 [71, 96]	79 [69, 91]	93 [76, 125.5]	<0.001
Potassium, mmol/L	2839	3.9 [3.6, 4.1]	3.9 [3.7, 4.1]	3.9 [3.6, 4.1]	3.9 [3.7, 4.2]	3.9 [3.6, 4.2]	0.808
C-reactive protein, mg/L	2748	1.4 [0.5, 7.0]	0.8 [0.4, 2.1]	1.4 [0.6, 6.1]	1.8 [0.6, 5.2]	8.9 [1.8, 42.8]	<0.001
ESR, mg/L	237	46.0 [23.0, 65.0]	46.0 [21.0, 63.0]	53.5 [27.2, 69.8]	69.0 [48.5, 82.0]	40.0 [21.5, 65.0]	0.481
Interleukin-6, mg/L	339	12.30 [5.45, 26.45]	5.00 [2.50, 12.50]	10.20 [5.40, 21.40]	14.30 [8.15, 58.15]	15.60 [8.22, 39.98]	<0.001
D-Dimer, mg/L	3004	0.320 [0.180, 0.650]	0.230 [0.145, 0.410]	0.320 [0.190, 0.610]	0.345 [0.195, 0.597]	0.660 [0.350, 1.525]	<0.001
Total cholesterol, mmol/L	2959	3.81 [3.17, 4.60]	3.90 [3.25, 4.72]	3.69 [3.07, 4.44]	4.20 [3.37, 5.31]	3.85 [3.20, 4.55]	<0.001
Total glyceride, mmol/L	2959	1.36 [0.97, 2.02]	1.35 [0.97, 2.05]	1.44 [1.04, 2.15]	1.33 [1.02, 1.74]	1.29 [0.93, 1.84]	<0.001
LDL-C, mmol/L	2959	1.94 [1.43, 2.63]	1.93 [1.44, 2.64]	1.83 [1.34, 2.51]	2.36 [1.74, 3.21]	2.09 [1.57, 2.77]	<0.001
Lactic acid, mmol/L	315	1.900 [1.480, 2.645]	1.690 [1.410, 2.160]	1.865 [1.435, 2.475]	1.800 [1.470, 2.330]	2.030 [1.520, 2.805]	0.159
Procalcitonin, mg/L	1141	0.048 [0.031, 0.088]	0.033 [0.022, 0.045]	0.046 [0.030, 0.070]	0.045 [0.032, 0.060]	0.080 [0.045, 0.201]	<0.001
Echocardiography findings							
LVEF, %	2141	64 [60, 67]	65 [63, 67]	64 [61, 67]	64 [53, 65.5]	58 [46, 64]	<0.001
LA, mm	2143	39 [36, 43]	38 [35, 41]	39 [36, 42]	45 [39, 48.5]	43 [38, 48]	<0.001
LVDd, mm	2143	47 [44, 50]	46 [43, 48]	47 [44, 50]	46 [44.5, 51.5]	48 [45, 54]	<0.001
PASP, mmHg	2097	31 [30, 35]	31 [30, 33]	31 [30, 34]	35 [31, 41.5]	33 [30, 39]	<0.001
IVST, mm	2140	10 [9, 11]	10 [9, 10]	10 [9, 12]	10 [9, 11]	11 [10, 12]	<0.001

*All patients with available data; AECOPD: Acute exacerbation chronic obstructive pulmonary disease; cTnT: Cardiac troponin T; NT-proBNP: N-terminal pro-B-type natriuretic peptide; ESR: Erythrocyte sedimentation rate; LDL-C: Low-density lipoprotein cholesterol; LVEF: Left Ventricular Ejection Fractions; LA: Left atrial diameter; LVDd: Left ventricular end-diastolic diameter; PASP: pulmonary systolic pressure; IVST: interventricular septal thickness.

**Table 2:** Outcomes stratified by cTNT & NT-proBNP

Outcomes		Overall	Normal cTnT & low NT-proBNP	Elevated cTnT & low NT-proBNP	Normal cTnT & high NT-proBNP	Elevated cTnT & high NT-proBNP
No. patients		N=2785	N=1180	N=908	N=38	N=659
Primary outcomes						
MACE (%)	Number of events (%)	232 (8.3)	37 (3.1)	73 (8.0)	2 (5.3)	120 (18.2)
	Adjusted HR (95%CI), P-value		Reference	2.47 (1.51-4.04), <0.001	3.89 (0.88-17.13), 0.073	2.85 (1.58-5.12), <0.001
Secondary outcomes						
All-cause death (%)	Number of events (%)	142 (5.1)	9 (0.8)	27 (3.0)	2 (5.3)	104 (15.8)
	Adjusted HR (95%CI), P-value		Reference	3.65 (1.02-13.10), 0.047	6.46 (0.63-66.04), 0.116	5.56 (1.51-20.52), 0.010
CV death (%)	Number of events (%)	69 (2.5)	4 (0.3)	15 (1.7)	1 (2.6)	49 (7.4)
	Adjusted HR (95%CI), P-value		Reference	9.64 (1.19-78.22), 0.034	26.90 (1.54-468.80), 0.024	11.97 (1.40-102.46), 0.023
CV-related rehospitalization (%)	Number of events (%)	185 (6.6)	34 (2.9)	66 (7.3)	1 (2.6)	84 (12.7)
	AMI (%)	49 (1.8)	8 (0.7)	28 (3.1)	0 (0.0)	13 (2.0)
	Stroke (%)	19 (0.7)	5 (0.4)	5 (0.6)	1 (2.6)	8 (1.2)
	Venous Thrombosis out (%)	4 (0.1)	0 (0.0)	1 (0.1)	0 (0.0)	3 (0.5)
	PTE (%)	2 (0.1)	0 (0.0)	0 (0.0)	0 (0.0)	2 (0.3)
	DVT (%)	2 (0.1)	0 (0.0)	1 (0.1)	0 (0.0)	1 (0.2)
	AHF (%)	117 (4.2)	22 (1.9)	33 (3.6)	0 (0.0)	62 (9.4)
	Adjusted HR (95%CI), P-value		Reference	2.24 (1.36-3.71), 0.002	2.20 (0.29-16.85), 0.446	2.38 (1.28-4.42), 0.006

MACE: a composited endpoint including at least one of the following endpoints: cardiovascular-related death, acute myocardial infarction, stroke, or acute heart failure (new-onset or worsening); CV death: cardiovascular-related death; CV-related rehospitalization: cardiovascular-related rehospitalization; AMI: acute myocardial infarction; PTE: pulmonary thromboembolism; DVT: deep vein thrombosis; AHF: acute heart failure (new-onset or worsening).

**Table 3:** In-hospital treatments and events of patients according to the level of cTnT and NT-proBNP

	Overall	Normal cTnT & low NT-proBNP	Elevated cTnT & low NT-proBNP	Normal cTnT & high NT-proBNP	Elevated cTnT & high NT-proBNP	P-Value
No. patients	N=3012	N=1283	N=975	N=48	N=706	
Medication treatmments						
Paxlovid (%)	3 (0.1)	0 (0.0)	1 (0.1)	0 (0.0)	2 (0.3)	0.147
Azvudine (%)	3 (0.1)	2 (0.2)	0 (0.0)	0 (0.0)	1 (0.1)	0.496
Glucocorticoid (%)	276 (9.2)	56 (4.4)	60 (6.2)	6 (12.5)	154 (21.8)	<0.001
Inotropes (%)	43 (1.4)	2 (0.2)	3 (0.3)	1 (2.1)	37 (5.2)	<0.001
Vasoactives (%)	157 (5.2)	5 (0.4)	22 (2.3)	2 (4.2)	128 (18.1)	<0.001
Oral anticoagulant (%)	398 (13.2)	139 (10.8)	77 (7.9)	24 (50.0)	158 (22.4)	<0.001
Enoxaparin sodium (%)	120 (4.0)	19 (1.5)	30 (3.1)	3 (6.2)	68 (9.6)	<0.001
Loop diuretic (%)	424 (14.1)	12 (0.9)	50 (5.1)	14 (29.2)	348 (49.3)	<0.001
Spironolactone (%)	477 (15.8)	35 (2.7)	72 (7.4)	26 (54.2)	344 (48.7)	<0.001
Digoxin (%)	91 (3.0)	12 (0.9)	14 (1.4)	13 (27.1)	52 (7.4)	<0.001
Aspirin (%)	977 (32.4)	420 (32.7)	429 (44.0)	4 (8.3)	124 (17.6)	<0.001
P2Y12 inhibitor (%)	1851 (61.5)	751 (58.5)	718 (73.6)	10 (20.8)	372 (52.7)	<0.001
Non-drug treatments						
Nasal cannula oxygen therapy (%)	740 (24.6)	118 (9.2)	179 (18.4)	14 (29.2)	429 (60.8)	<0.001
Mask oxygen therapy (%)	19 (0.6)	1 (0.1)	1 (0.1)	0 (0.0)	17 (2.4)	<0.001
Invasive mechanical ventilation (%)	91 (3.0)	0 (0.0)	8 (0.8)	1 (2.1)	82 (11.6)	<0.001
ECMO (%)	3 (0.1)	0 (0.0)	1 (0.1)	0 (0.0)	2 (0.3)	0.147
IABP (%)	1 (0.0)	0 (0.0)	0 (0.0)	0 (0.0)	1 (0.1)	0.250
Events						
Hypoxemia	16 (0.5)	2 (0.2)	1 (0.1)	0 (0.0)	13 (1.8)	<0.001
Respiratory failure	41 (1.4)	1 (0.1)	5 (0.5)	1 (2.1)	34 (4.8)	<0.001
Severe pneumonia	11 (0.4)	1 (0.1)	2 (0.2)	0 (0.0)	8 (1.1)	0.005
Sepsis	2 (0.1)	0 (0.0)	0 (0.0)	0 (0.0)	2 (0.3)	0.087
Cardiac arrest	4 (0.1)	0 (0.0)	2 (0.2)	0 (0.0)	2 (0.3)	0.214
Shock	9 (0.3)	0 (0.0)	0 (0.0)	0 (0.0)	9 (1.3)	<0.001
Death in-hospital	66 (2.2)	0 (0.0)	6 (0.6)	1 (2.1)	59 (8.4)	<0.001

ECMO: Extracorporeal membrane oxygenation; IABP: Intra-aortic balloon pump.

## Abbreviations

ACS: acute coronary syndrome
AMI: acute myocardial infarction
BMI: body mass index
CAD: coronary artery disease
COVID-19: coronavirus disease 2019
CRP: C-reactive protein
cTnT: cardiac troponins T
cTnI: cardiac troponins I
CV: cardiovascular
DVT: deep vein thrombosis
HF: heart failure
MACEs: major adverse cardiac events
NT-proBNP: N-terminal pro-B-type natriuretic peptide
PTE: pulmonary thromboembolism
SARS-CoV-2: severe acute respiratory syndrome coronavirus 2

## References

[1] World Health Organization. WHO coronavirus (COVID-19) dashboard. 2024.

[2] Bowe B, Xie Y, Xu E, Al-Aly Z (2021). Kidney Outcomes in Long COVID. J Am Soc Nephrol.

[3] DeVries A, Shambhu S, Sloop S, Overhage JM (2023). One-Year Adverse Outcomes Among US Adults With Post-COVID-19 Condition vs Those Without COVID-19 in a Large Commercial Insurance Database. JAMA Health Forum.

[4] Wan EYF, Mathur S, Zhang R, Yan VKC, Lai FTT, Chui CSL (2023). Association of COVID-19 with short- and long-term risk of cardiovascular disease and mortality: a prospective cohort in UK Biobank. Cardiovasc Res.

[5] Xie Y, Xu E, Bowe B, Al-Aly Z (2022). Long-term cardiovascular outcomes of COVID-19. Nat Med.

[6] Lu G, Zhang Y, Zhang H, Ai J, He L, Yuan X (2022). Geriatric risk and protective factors for serious COVID-19 outcomes among older adults in Shanghai Omicron wave. Emerg Microbes Infect.

[7] Wu Q, Wang H, Cai J, Ai J, Li Y, Zhang H (2023). Vaccination effects on post-infection outcomes in the Omicron BA.2 outbreak in Shanghai. Emerg Microbes Infect.

[8] Nyberg T, Ferguson NM, Nash SG, Webster HH, Flaxman S, Andrews N (2022). Comparative analysis of the risks of hospitalisation and death associated with SARS-CoV-2 omicron (B.1.1.529) and delta (B.1.617.2) variants in England: a cohort study. Lancet.

[9] Menni C, Valdes AM, Polidori L, Antonelli M, Penamakuri S, Nogal A (2022). Symptom prevalence, duration, and risk of hospital admission in individuals infected with SARS-CoV-2 during periods of omicron and delta variant dominance: a prospective observational study from the ZOE COVID Study. Lancet.

[10] Yuan S, Ye ZW, Liang R, Tang K, Zhang AJ, Lu G (2022). Pathogenicity, transmissibility, and fitness of SARS-CoV-2 Omicron in Syrian hamsters. Science.

[11] Shi S, Qin M, Shen B, Cai Y, Liu T, Yang F (2020). Association of Cardiac Injury With Mortality in Hospitalized Patients With COVID-19 in Wuhan, China. JAMA Cardiol.

[12] Shi X, Chen M, Zhang Y (2021). The cardiovascular disorders and prognostic cardiac biomarkers in COVID-19. Mol Biol Rep.

[13] O'Donnell C, Ashland MD, Vasti EC, Lu Y, Chang AY, Wang P (2021). N-Terminal Pro-B-Type Natriuretic Peptide as a Biomarker for the Severity and Outcomes With COVID-19 in a Nationwide Hospitalized Cohort. J Am Heart Assoc.

[14] Sabanoglu C, Inanc IH, Polat E, Peker SA (2022). Long-term predictive value of cardiac biomarkers in patients with COVID-19 infection. Eur Rev Med Pharmacol Sci.

[15] Cunningham JW, Claggett BL, Jering KS, Vaduganathan M, Bhatt AS, Rosenthal N, Solomon SD (2021). Prognostic Value of Natriuretic Peptides and Cardiac Troponins in COVID-19. Circulation.

[16] Maron DJ, Hochman JS, Reynolds HR, Bangalore S, O'Brien SM, Boden WE (2020). Initial Invasive or Conservative Strategy for Stable Coronary Disease. N Engl J Med.

[17] Manocha KK, Kirzner J, Ying X, Yeo I, Peltzer B, Ang B (2021). Troponin and Other Biomarker Levels and Outcomes Among Patients Hospitalized With COVID-19: Derivation and Validation of the HA2T2 COVID-19 Mortality Risk Score. J Am Heart Assoc.

[18] Stefanini GG, Chiarito M, Ferrante G, Cannata F, Azzolini E, Viggiani G (2020). Early detection of elevated cardiac biomarkers to optimise risk stratification in patients with COVID-19. Heart.

[19] Putter H, Schumacher M, van Houwelingen HC (2020). On the relation between the cause-specific hazard and the subdistribution rate for competing risks data: The Fine-Gray model revisited. Biom J.

[20] Tromp J, Paniagua SMA, Lau ES, Allen NB, Blaha MJ, Gansevoort RT (2021). Age dependent associations of risk factors with heart failure: pooled population based cohort study. BMJ.

[21] Jepma P, Jorstad HT, Snaterse M, Ter Riet G, Kragten H, Lachman S (2020). Lifestyle modification in older versus younger patients with coronary artery disease. Heart.

[22] Warren-Gash C, Davidson JA, Strongman H, Herrett E, Smeeth L, Breuer J, Banerjee A (2023). Severe COVID-19 outcomes by cardiovascular risk profile in England in 2020: a population-based cohort study. Lancet Reg Health Eur.

[23] Reyes LF, Garcia-Gallo E, Murthy S, Fuentes YV, Serrano CC, Ibanez-Prada ED (2023). Major adverse cardiovascular events (MACE) in patients with severe COVID-19 registered in the ISARIC WHO clinical characterization protocol: A prospective, multinational, observational study. J Crit Care.

[24] Tamura T, Mizuma K, Nasser H, Deguchi S, Padilla-Blanco M, Oda Y (2024). Virological characteristics of the SARS-CoV-2 BA.2.86 variant. Cell Host Microbe.

[25] Qu P, Xu K, Faraone JN, Goodarzi N, Zheng YM, Carlin C (2024). Immune evasion, infectivity, and fusogenicity of SARS-CoV-2 BA.2.86 and FLip variants. Cell.

[26] Tajbakhsh A, Gheibi Hayat SM, Taghizadeh H, Akbari A, Inabadi M, Savardashtaki A (2021). COVID-19 and cardiac injury: clinical manifestations, biomarkers, mechanisms, diagnosis, treatment, and follow up. Expert Rev Anti Infect Ther.

[27] Fish-Trotter H, Ferguson JF, Patel N, Arora P, Allen NB, Bachmann KN (2020). Inflammation and Circulating Natriuretic Peptide Levels. Circ Heart Fail.

[28] Qin JJ, Cheng X, Zhou F, Lei F, Akolkar G, Cai J (2020). Redefining Cardiac Biomarkers in Predicting Mortality of Inpatients With COVID-19. Hypertension.

[29] Wang W, Wang CY, Wang SI, Wei JC (2022). Long-term cardiovascular outcomes in COVID-19 survivors among non-vaccinated population: A retrospective cohort study from the TriNetX US collaborative networks. EClinicalMedicine.

[30] Giannitsis E, Katus HA (2013). Cardiac troponin level elevations not related to acute coronary syndromes. Nat Rev Cardiol.

[31] Majure DT, Gruberg L, Saba SG, Kvasnovsky C, Hirsch JS, Jauhar R, Northwell Health C-RC (2021). Usefulness of Elevated Troponin to Predict Death in Patients With COVID-19 and Myocardial Injury. Am J Cardiol.

[32] Goetze JP, Bruneau BG, Ramos HR, Ogawa T, de Bold MK, de Bold AJ (2020). Cardiac natriuretic peptides. Nat Rev Cardiol.

[33] Guo T, Fan Y, Chen M, Wu X, Zhang L, He T (2020). Cardiovascular Implications of Fatal Outcomes of Patients With Coronavirus Disease 2019 (COVID-19). JAMA Cardiol.

[34] Caro-Codon J, Rey JR, Buno A, Iniesta AM, Rosillo SO, Castrejon-Castrejon S (2021). Characterization of NT-proBNP in a large cohort of COVID-19 patients. Eur J Heart Fail.

[35] Lombardi CM, Carubelli V, Iorio A, Inciardi RM, Bellasi A, Canale C (2020). Association of Troponin Levels With Mortality in Italian Patients Hospitalized With Coronavirus Disease 2019: Results of a Multicenter Study. JAMA Cardiol.

