# Supplementary material for: Long-term Cardiovascular Outcomes in Patients with Omicron COVID-19 and Elevated Cardiac Biomarkers: A Prospective Multicenter Cohort Study in Shanghai, China
Source: Int J Med Sci. 2025 Jun 9;22(12):2884–95. doi: 10.7150/ijms.112282 (PMC12243857; doi:10.7150/ijms.112282)
Supplement: Supplementary file 1 — Supplementary figures and tables. [file ijmsv22p2884s1.pdf]

Long-term Cardiovascular Outcomes in Patients with Omicron COVID-19 and Elevated Cardiac Biomarkers: A Prospective Multicenter Cohort Study in Shanghai, China

Supplementary tables and figures:

Table S1. Outcomes Stratified by cTnT & NT-proBNP among Patients Discharged Alive 2

Table S2. Incidence of events stratified by cTNT & NT-proBNP in subgroups 3

Figure S1. The diagram illustrates the flow of patient discharged alive inclusion in this cohort. 7

Figure S2. Kaplan–Meier curves stratified by the level of cTnT and NT-proBNP in patients discharged alive for the cumulative incidence 8

Figure S3. Kaplan–Meier curves stratified by the level of cTnT and NT-proBNP for the cumulative incidence among patients with ACS or HF 9

Figure S4. Kaplan–Meier curves stratified by the level of cTnT and NT-proBNP for the cumulative incidence among patients without ACS or HF 10

Figure S5. Subgroup analysis by CRP level for MACEs in the overall cohort. 11

Table S1. Outcomes Stratified by cTnT & NT-proBNP among Patients Discharged Alive

Outcomes		Overall	Normal cTnT & low NT-proBNP	Elevated cTnT & low NT-proBNP	Normal cTnT & high NT-proBNP	Elevated cTnT & high NT-proBNP
No. patients		N=2719	N=1180	N=902	N=37	N=600
Primary outcomes						
MACEs (%)	Number of events (%)	211 (7.8)	37 (3.1)	72 (8.0)	2 (5.4)	100 (16.7)
	Adjusted HR (95%CI), P-value		Reference	2.48(1.51, 4.06), <0.001	3.89 (0.88, 17.20), 0.073	2.58 (1.42, 4.70), 0.002
Secondary outcomes						
All-cause death (%)	Number of events (%)	76 (2.8)	9 (0.8)	21 (2.3)	1 (2.7)	45 (7.5)
	Adjusted HR (95%CI), P-value		Reference	3.33 (0.89, 12.40), 0.073	8.73 (0.83, 91.52), 0.071	4.15 (1.05, 16.32), 0.042
CV death (%)	Number of events (%)	48 (1.8)	4 (0.3)	14 (1.6)	1 (2.7)	29 (4.8)
	Adjusted HR (95%CI), P-value		Reference	9.56 (1.17, 78.33), 0.035	21.93 (1.22, 393.74), 0.036	7.02 (0.78, 63.02), 0.082
CV-related rehospitalization (%)	Number of events (%)	185 (6.8)	34 (2.9)	66 (7.3)	1 (2.7)	84 (14.0)
	AMI (%)	49 (1.8)	8 (0.7)	28 (3.1)	0 (0.0)	13 (2.2)
	Stroke (%)	19 (0.7)	5 (0.4)	5 (0.6)	1 (2.7)	8 (1.3)
	Venous Thrombosis (%)	4 (0.1)	0 (0.0)	1 (0.1)	0 (0.0)	3 (0.5)
	PTE (%)	2 (0.1)	0 (0.0)	0 (0.0)	0 (0.0)	2 (0.3)
	DVT (%)	2 (0.1)	0 (0.0)	1 (0.1)	0 (0.0)	1 (0.2)
	AHF (%)	117 (4.3)	22 (1.9)	33 (3.7)	0 (0.0)	62 (10.3)
	Adjusted HR (95%CI), P-value		Reference	2.24 (1.36, 3.71), 0.002	2.20 (0.29-16.84), 0.447	2.38 (1.28-4.42), 0.006

Data are n (%);HR: Hazard ratios; 95%CI :95% confidence intervals. MACEs: a composited endpoint including at least one of the following endpoints: cardiovascular-related death, acute myocardial infarction, stroke, or acute heart failure (new-onset or worsening); CV death: cardiovascular-related death; CV-related rehospitalization: cardiovascular-related rehospitalization; AMI: acute myocardial infarction; PTE: pulmonary thromboembolism; DVT: deep vein thrombosis; AHF: acute heart failure (new-onset or worsening).

Table S2. Incidence of events stratified by cTNT & NT-proBNP in subgroups

Outcomes	Normal cTnT & low NT-proBNP	Elevated cTnT & low NT-proBNP	Normal cTnT & high NT-proBNP	Elevated cTnT & high NT-proBNP	P-Value
**Age**					
**<65 yr**	680	377	17	152	
MACEs (%)	18 (2.6)	30 (8.0)	0 (0.0)	19 (12.5)	<0.001
All-cause death (%)	1 (0.1)	5 (1.3)	0 (0.0)	7 (4.6)	<0.001
CV death (%)	1 (0.1)	5 (1.3)	0 (0.0)	5 (3.3)	0.002
CV-related rehospitalization (%)	17 (2.5)	26 (6.9)	0 (0.0)	15 (9.9)	<0.001
AMI (%)	3 (0.4)	10 (2.7)	0 (0.0)	4 (2.6)	0.01
Stroke (%)	3 (0.4)	2 (0.5)	0 (0.0)	0 (0.0)	1
Venous Thrombosis (%)	0 (0.0)	1 (0.3)	0 (0.0)	0 (0.0)	0.45
PTE (%)	0 (0.0)	0 (0.0)	0 (0.0)	0 (0.0)	NA
DVT (%)	0 (0.0)	1 (0.3)	0 (0.0)	0 (0.0)	0.45
AHF (%)	11 (1.6)	13 (3.4)	0 (0.0)	11 (7.2)	0.004
**≥65 yr**	500	531	21	507	
MACEs (%)	19 (3.8)	43 (8.1)	2 (9.5)	101 (19.9)	<0.001
All-cause death (%)	8 (1.6)	22 (4.1)	2 (9.5)	97 (19.1)	<0.001
CV death (%)	3 (0.6)	10 (1.9)	1 (4.8)	44 (8.7)	<0.001
CV-related rehospitalization (%)	17 (3.4)	40 (7.5)	1 (4.8)	69 (13.6)	<0.001
AMI (%)	5 (1.0)	18 (3.4)	0 (0.0)	9 (1.8)	0.06
Stroke (%)	2 (0.4)	3 (0.6)	1 (4.8)	8 (1.6)	0.05
Venous Thrombosis (%)	0 (0.0)	0 (0.0)	0 (0.0)	3 (0.6)	0.11
PTE (%)	0 (0.0)	0 (0.0)	0 (0.0)	2 (0.4)	0.24
DVT (%)	0 (0.0)	0 (0.0)	0 (0.0)	1 (0.2)	0.66
AHF (%)	11 (2.2)	20 (3.8)	0 (0.0)	51 (10.1)	<0.001
**Sex**					
**Female**	528	198	20	219	
MACEs (%)	14 (2.7)	16 (8.1)	2 (10.0)	46 (21.0)	<0.001
All-cause death (%)	6 (1.1)	7 (3.5)	1 (5.0)	35 (16.0)	<0.001
CV death (%)	2 (0.4)	3 (1.5)	1 (5.0)	18 (8.2)	<0.001
CV-related rehospitalization (%)	13 (2.5)	15 (7.6)	1 (5.0)	34 (15.5)	<0.001
AMI (%)	3 (0.6)	3 (1.5)	0 (0.0)	7 (3.2)	0.04
Stroke (%)	1 (0.2)	0 (0.0)	1 (5.0)	3 (1.4)	0.02
Venous Thrombosis (%)	0 (0.0)	0 (0.0)	0 (0.0)	1 (0.5)	0.45
PTE (%)	0 (0.0)	0 (0.0)	0 (0.0)	1 (0.5)	0.45
DVT (%)	0 (0.0)	0 (0.0)	0 (0.0)	0 (0.0)	NA
AHF (%)	9 (1.7)	13 (6.6)	0 (0.0)	23 (10.5)	<0.001

Table S2. Continued

**Male**	652	710	18	440	
MACEs (%)	23 (3.5)	57 (8.0)	0 (0.0)	74 (16.8)	<0.001
All-cause death (%)	3 (0.5)	20 (2.8)	1 (5.6)	69 (15.7)	<0.001
CV death (%)	2 (0.3)	12 (1.7)	0 (0.0)	31 (7.0)	<0.001
CV-related rehospitalization (%)	21 (3.2)	51 (7.2)	0 (0.0)	50 (11.4)	<0.001
AMI (%)	5 (0.8)	25 (3.5)	0 (0.0)	6 (1.4)	0.003
Stroke (%)	4 (0.6)	5 (0.7)	0 (0.0)	5 (1.1)	0.67
Venous Thrombosis (%)	0 (0.0)	1 (0.1)	0 (0.0)	2 (0.5)	0.28
PTE (%)	0 (0.0)	0 (0.0)	0 (0.0)	1 (0.2)	0.25
DVT (%)	0 (0.0)	1 (0.1)	0 (0.0)	1 (0.2)	0.72
AHF (%)	13 (2.0)	20 (2.8)	0 (0.0)	39 (8.9)	<0.001
**Hypertension**					
**No**	594	302	20	285	
MACEs (%)	13 (2.2)	25 (8.3)	0 (0.0)	50 (17.5)	<0.001
All-cause death (%)	6 (1.0)	9 (3.0)	1 (5.0)	43 (15.1)	<0.001
CV death (%)	1 (0.2)	7 (2.3)	0 (0.0)	21 (7.4)	<0.001
CV-related rehospitalization (%)	12 (2.0)	20 (6.6)	0 (0.0)	37 (13.0)	<0.001
AMI (%)	2 (0.3)	11 (3.6)	0 (0.0)	4 (1.4)	0.002
Stroke (%)	1 (0.2)	1 (0.3)	0 (0.0)	4 (1.4)	0.12
Venous Thrombosis (%)	0 (0.0)	0 (0.0)	0 (0.0)	1 (0.4)	0.25
PTE (%)	0 (0.0)	0 (0.0)	0 (0.0)	0 (0.0)	NA
DVT (%)	0 (0.0)	0 (0.0)	0 (0.0)	1 (0.4)	0.25
AHF (%)	9 (1.5)	8 (2.6)	0 (0.0)	29 (10.2)	<0.001
**Yes**	586	606	18	374	
MACEs (%)	24 (4.1)	48 (7.9)	2 (11.1)	70 (18.7)	<0.001
All-cause death (%)	3 (0.5)	18 (3.0)	1 (5.6)	61 (16.3)	<0.001
CV death (%)	3 (0.5)	8 (1.3)	1 (5.6)	28 (7.5)	<0.001
CV-related rehospitalization (%)	22 (3.8)	46 (7.6)	1 (5.6)	47 (12.6)	<0.001
AMI (%)	6 (1.0)	17 (2.8)	0 (0.0)	9 (2.4)	0.14
Stroke (%)	4 (0.7)	4 (0.7)	1 (5.6)	4 (1.1)	0.2
Venous Thrombosis (%)	0 (0.0)	1 (0.2)	0 (0.0)	2 (0.5)	0.28
PTE (%)	0 (0.0)	0 (0.0)	0 (0.0)	2 (0.5)	0.08
DVT (%)	0 (0.0)	1 (0.2)	0 (0.0)	0 (0.0)	1
AHF (%)	13 (2.2)	25 (4.1)	0 (0.0)	33 (8.8)	<0.001
**CAD**					
**No**	630	204	33	295	
MACEs (%)	12 (1.9)	12 (5.9)	1 (3.0)	53 (18.0)	<0.001
All-cause death (%)	7 (1.1)	12 (5.9)	1 (3.0)	52 (17.6)	<0.001
CV death (%)	3 (0.5)	4 (2.0)	0 (0.0)	23 (7.8)	<0.001

Table S2. Continued

All-cause rehospitalization (%)	43 (6.8)	31 (15.2)	2 (6.1)	56 (19.0)	<0.001
CV-related rehospitalization (%)	10 (1.6)	10 (4.9)	1 (3.0)	38 (12.9)	<0.001
AMI (%)	2 (0.3)	3 (1.5)	0 (0.0)	0 (0.0)	0.12
Stroke (%)	1 (0.2)	2 (1.0)	1 (3.0)	4 (1.4)	0.03
Venous Thrombosis (%)	0 (0.0)	0 (0.0)	0 (0.0)	3 (1.0)	0.04
PTE (%)	0 (0.0)	0 (0.0)	0 (0.0)	2 (0.7)	0.15
DVT (%)	0 (0.0)	0 (0.0)	0 (0.0)	1 (0.3)	0.46
AHF (%)	8 (1.3)	5 (2.5)	0 (0.0)	31 (10.5)	<0.001
**Yes**	550	704	5	364	
MACEs (%)	25 (4.5)	61 (8.7)	1 (20.0)	67 (18.4)	<0.001
All-cause death (%)	2 (0.4)	15 (2.1)	1 (20.0)	52 (14.3)	<0.001
CV death (%)	1 (0.2)	11 (1.6)	1 (20.0)	26 (7.1)	<0.001
CV-related rehospitalization (%)	24 (4.4)	56 (8.0)	0 (0.0)	46 (12.6)	<0.001
AMI (%)	6 (1.1)	25 (3.6)	0 (0.0)	13 (3.6)	0.02
Stroke (%)	4 (0.7)	3 (0.4)	0 (0.0)	4 (1.1)	0.42
Venous Thrombosis (%)	0 (0.0)	1 (0.1)	0 (0.0)	0 (0.0)	1
PTE (%)	0 (0.0)	0 (0.0)	0 (0.0)	0 (0.0)	NA
DVT (%)	0 (0.0)	1 (0.1)	0 (0.0)	0 (0.0)	1
AHF (%)	14 (2.5)	28 (4.0)	0 (0.0)	31 (8.5)	0
**ACS/HF**					
**No**	1040	590	25	293	
MACEs (%)	32 (3.1)	46 (7.8)	1 (4.0)	36 (12.3)	<0.001
All-cause death (%)	9 (0.9)	20 (3.4)	1 (4.0)	45 (15.4)	<0.001
CV death (%)	4 (0.4)	10 (1.7)	0 (0.0)	11 (3.8)	<0.001
CV-related rehospitalization (%)	29 (2.8)	42 (7.1)	1 (4.0)	28 (9.6)	<0.001
AMI (%)	7 (0.7)	16 (2.7)	0 (0.0)	5 (1.7)	0.01
Stroke (%)	5 (0.5)	5 (0.8)	1 (4.0)	5 (1.7)	0.05
Venous Thrombosis (%)	0 (0.0)	1 (0.2)	0 (0.0)	2 (0.7)	0.06
PTE (%)	0 (0.0)	0 (0.0)	0 (0.0)	1 (0.3)	0.16
DVT (%)	0 (0.0)	1 (0.2)	0 (0.0)	1 (0.3)	0.14
AHF (%)	18 (1.7)	21 (3.6)	0 (0.0)	18 (6.1)	0
**Yes**	140	318	13	366	
MACEs (%)	5 (3.6)	27 (8.5)	1 (7.7)	84 (23.0)	<0.001
All-cause death (%)	0 (0.0)	7 (2.2)	1 (7.7)	59 (16.1)	<0.001
CV death (%)	0 (0.0)	5 (1.6)	1 (7.7)	38 (10.4)	<0.001
CV-related rehospitalization (%)	5 (3.6)	24 (7.5)	0 (0.0)	56 (15.3)	<0.001
AMI (%)	1 (0.7)	12 (3.8)	0 (0.0)	8 (2.2)	0.28
Stroke (%)	0 (0.0)	0 (0.0)	0 (0.0)	3 (0.8)	0.33
Venous Thrombosis (%)	0 (0.0)	0 (0.0)	0 (0.0)	1 (0.3)	1

Table S2. Continued

PTE (%)	0 (0.0)	0 (0.0)	0 (0.0)	1 (0.3)	1
DVT (%)	0 (0.0)	0 (0.0)	0 (0.0)	0 (0.0)	NA
AHF (%)	4 (2.9)	12 (3.8)	0 (0.0)	44 (12.0)	<0.001
**CRP**					
**< 3.0 mg/L**	855	531	19	202	
MACEs (%)	27 (3.2)	40 (7.5)	1 (5.3)	30 (14.9)	<0.001
All-cause death (%)	2 (0.2)	5 (0.9)	0 (0.0)	10 (5.0)	<0.001
CV death (%)	1 (0.1)	4 (0.8)	0 (0.0)	5 (2.5)	0.004
CV-related rehospitalization (%)	27 (3.2)	39 (7.3)	1 (5.3)	28 (13.9)	<0.001
AMI (%)	6 (0.7)	19 (3.6)	0 (0.0)	4 (2.0)	0.002
Stroke (%)	3 (0.4)	4 (0.8)	1 (5.3)	3 (1.5)	0.078
Venous Thrombosis (%)	0 (0.0)	1 (0.2)	0 (0.0)	1 (0.5)	0.441
PTE (%)	0 (0.0)	0 (0.0)	0 (0.0)	0 (0.0)	NA
DVT (%)	0 (0.0)	1 (0.2)	0 (0.0)	1 (0.5)	0.122
AHF (%)	19 (2.2)	15 (2.8)	0 (0.0)	21 (10.4)	<0.001
**≥ 3.0 mg/L**	197	298	15	416	
MACEs (%)	7 (3.6)	27 (9.1)	1 (6.7)	85 (20.4)	<0.001
All-cause death (%)	6 (3.0)	21 (7.0)	2 (13.3)	89 (21.4)	<0.001
CV death (%)	2 (1.0)	10 (3.4)	1 (6.7)	43 (10.3)	<0.001
CV-related rehospitalization (%)	5 (2.5)	21 (7.0)	0 (0.0)	52 (12.5)	<0.001
AMI (%)	1 (0.5)	6 (2.0)	0 (0.0)	7 (1.7)	0.539
Stroke (%)	1 (0.5)	0 (0.0)	0 (0.0)	5 (1.2)	0.19
Venous Thrombosis (%)	0 (0.0)	0 (0.0)	0 (0.0)	2 (0.5)	0.711
PTE (%)	0 (0.0)	0 (0.0)	0 (0.0)	2 (0.5)	0.711
DVT (%)	0 (0.0)	0 (0.0)	0 (0.0)	0 (0.0)	NA
AHF (%)	3 (1.5)	16 (5.4)	0 (0.0)	39 (9.4)	<0.001

Data are n (%). MACEs: a composited endpoint including at least one of the following endpoints: cardiovascular-related death, acute myocardial infarction, stroke, or acute heart failure (new-onset or worsening); CV death: cardiovascular-related death; CV-related rehospitalization: cardiovascular-related rehospitalization; AMI: acute myocardial infarction; PTE: pulmonary thromboembolism; DVT: deep vein thrombosis; AHF: acute heart failure (new-onset or worsening); NA: not available.

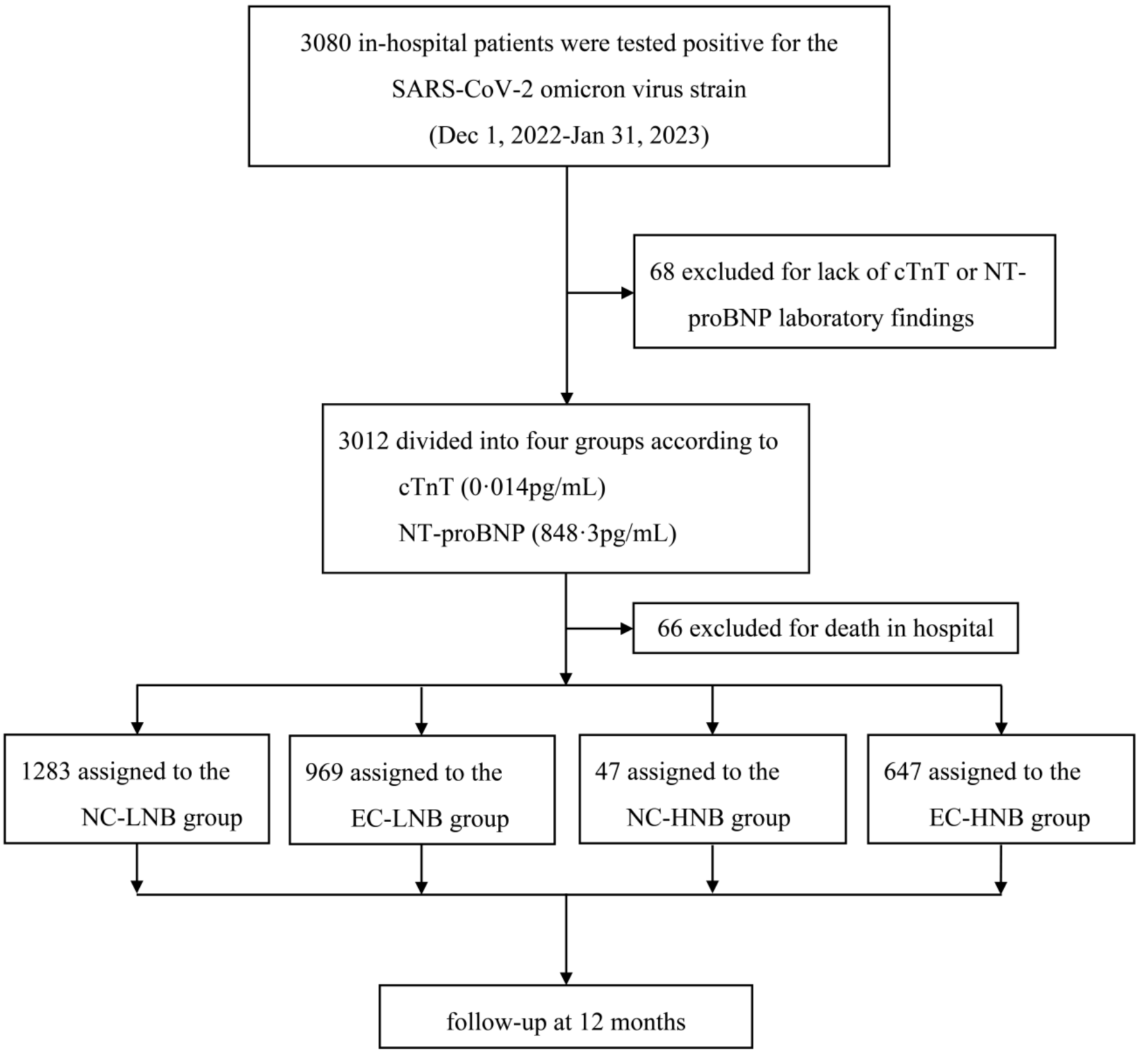

Figure S1. The diagram illustrates the flow of patient discharged alive inclusion in this cohort. The NC-LNB group, the normal cTnT and low NT-proBNP group; The EC-LNB group, the elevated cTnT and low NT-proBNP group; The NC-HNB group, the normal cTnT and high NT-proBNP group; The EC-HNB group, the elevated cTnT and high NT-proBNP group. cTnT, cardiac troponin-T; NT-proBNP, N-terminal pro-B-type natriuretic peptide.

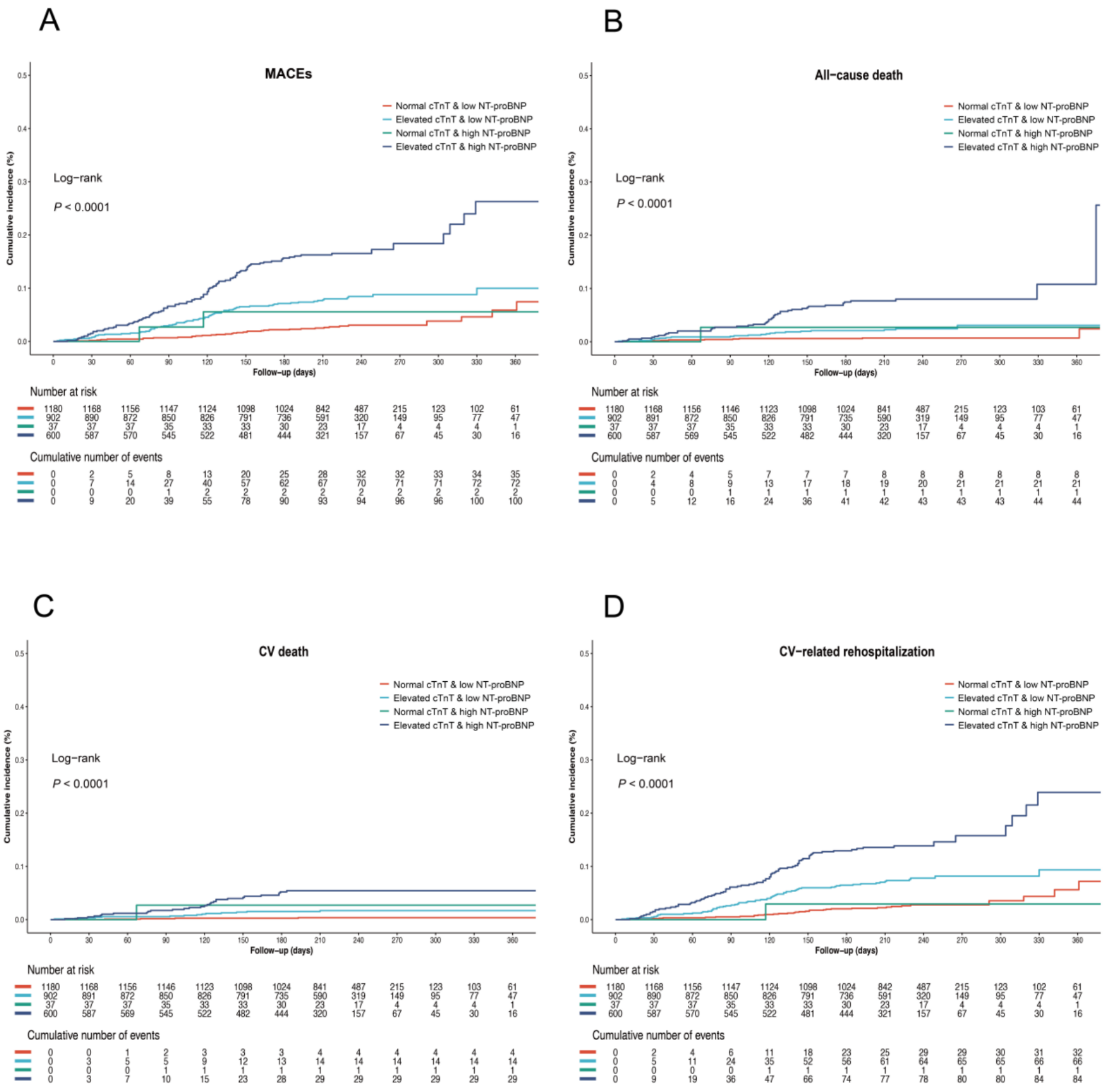

Figure S2. Kaplan–Meier curves stratified by the level of cTnT and NT-proBNP for the cumulative incidence of (A) MACEs, (B) all-cause death, (C) cardiovascular death, and (D) cardiovascular-related rehospitalization among patients who were discharged alive. MACEs, major adverse cardiovascular events.

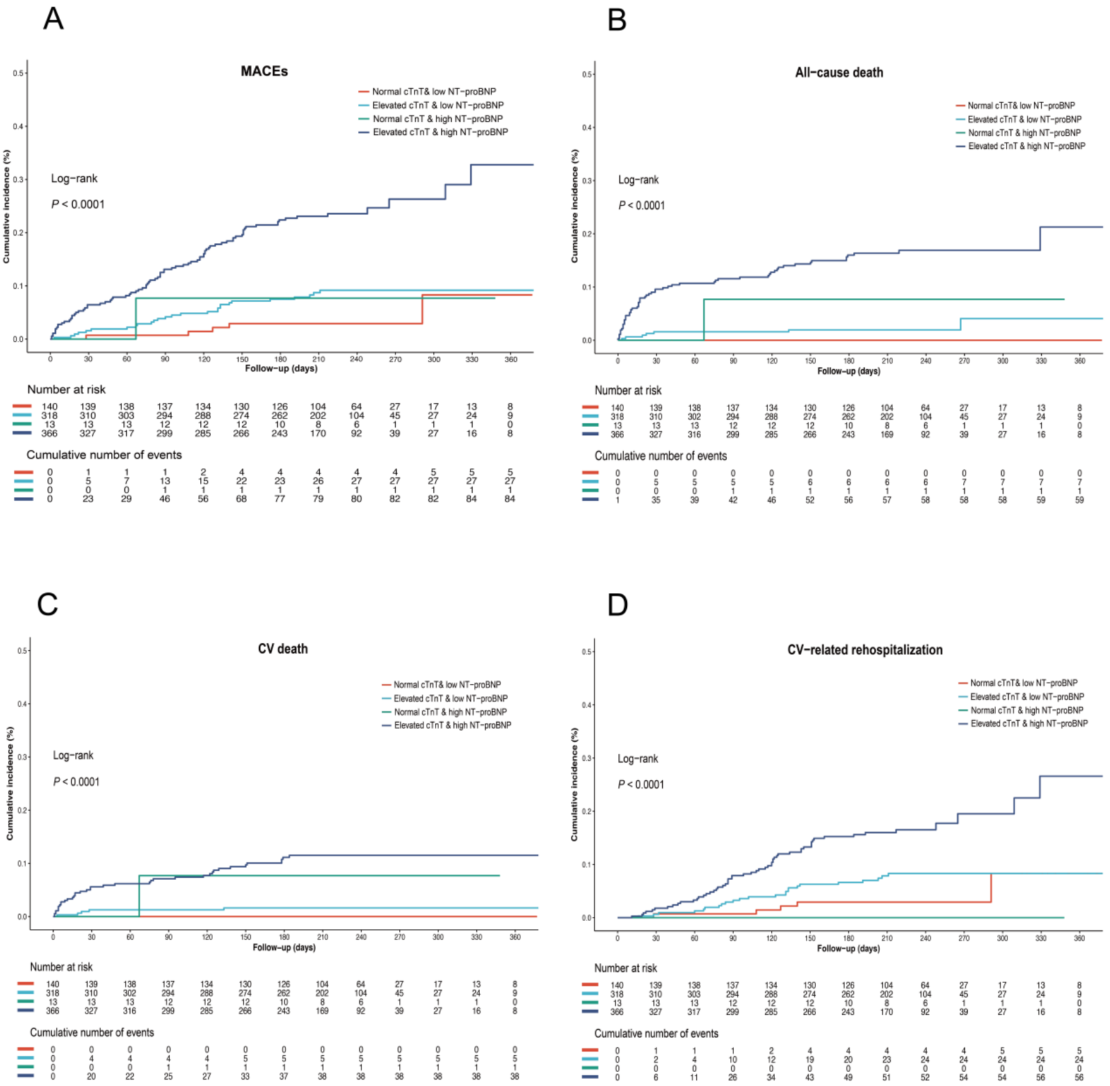

Figure S3. Kaplan–Meier curves stratified by the level of cTnT and NT-proBNP for the cumulative incidence of (A) MACEs, (B) all-cause death, (C) cardiovascular death, and (D) cardiovascular-related rehospitalization among patients with acute coronary syndrome (ACS) or heart failure (HF). MACEs, major adverse cardiovascular events.

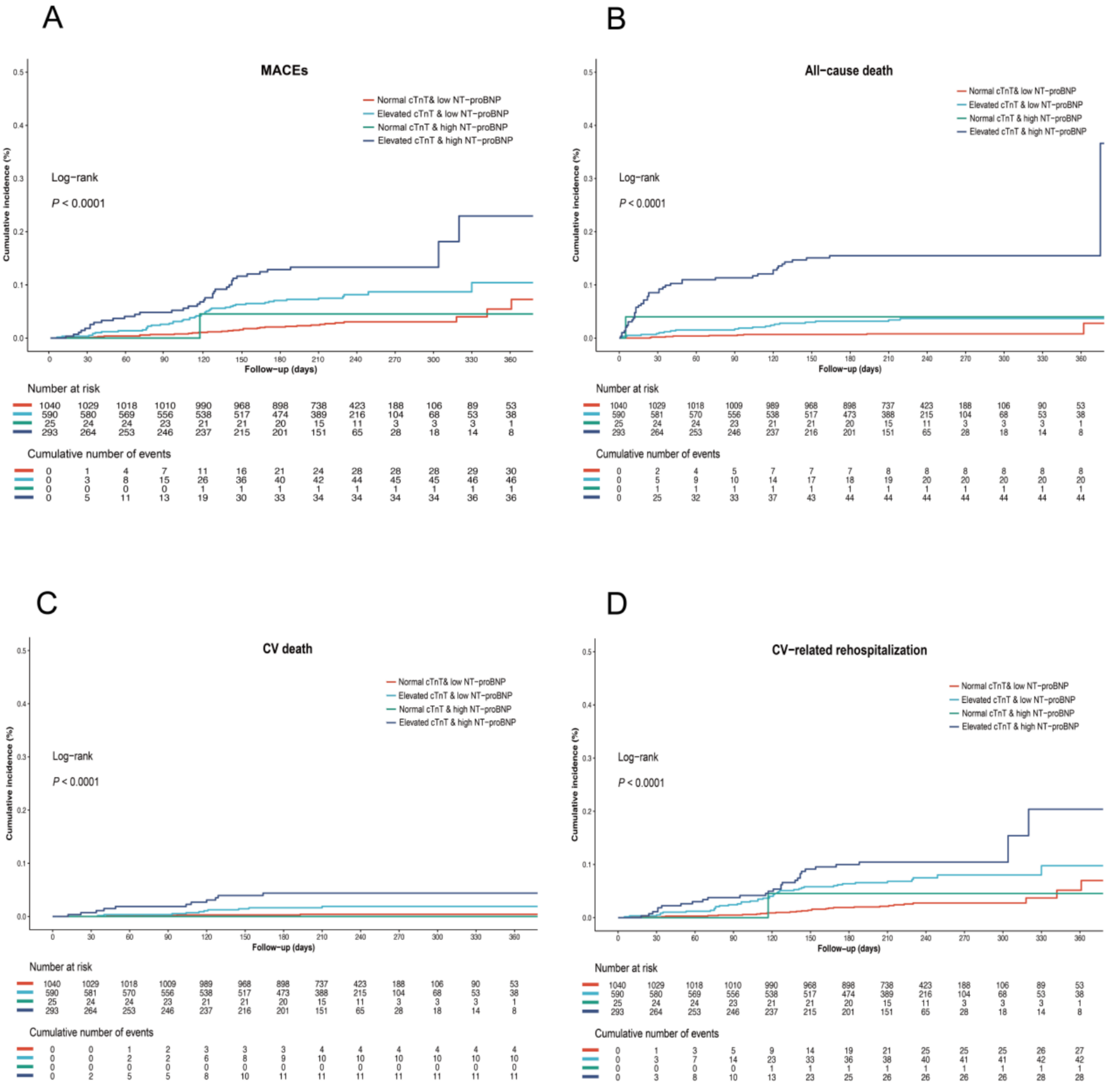

Figure S4. Kaplan–Meier curves stratified by the level of cTnT and NT-proBNP for the cumulative incidence of (A) MACEs, (B) all-cause death, (C) cardiovascular death, and (D) cardiovascular-related rehospitalization among patients without ACS or HF. MACEs, major adverse cardiovascular events.

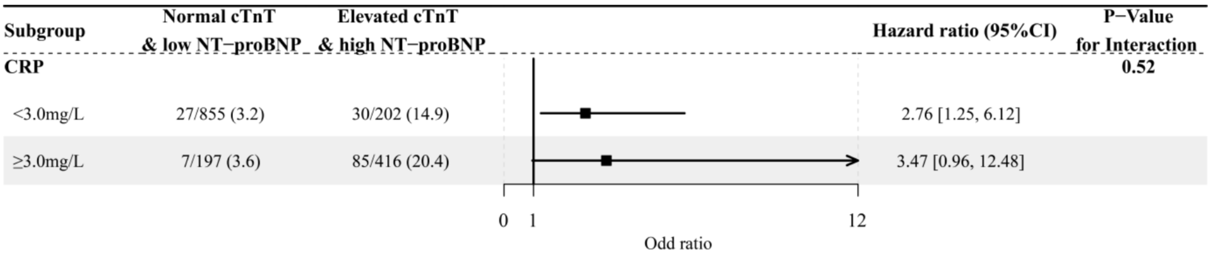

Figure S5. Subgroup analysis by CRP level for MACEs in the overall cohort. Based on the upper limit of normal for CRP (3.0 mg/L); CRP, C reactive protein; HR, hazard ratio.
